# Pseudophakic pupillary block after reverse sulcus intraocular lens implantation: A case report

**DOI:** 10.1097/MD.0000000000033496

**Published:** 2023-04-14

**Authors:** Panrapee Funarunart, Isaraporn Treesit

**Affiliations:** a Department of Ophthalmology, Phramongkutklao Hospital, Phramongkutklao College of Medicine, Bangkok, Thailand.

**Keywords:** case report, pseudophakic, pupillary block, pupillary capture, reverse sulcus intraocular lens

## Abstract

Reverse sulcus intraocular lens (IOL) implantation can cause serious ophthalmic complications. Even routine ocular examinations can provoke significant consequences. Herein, we present a case of recurrent pupillary capture and pseudophakic pupillary block related to reverse sulcus IOL implantation.

**Case report:**

A 44-year-old man developed a pseudophakic pupillary block after pupil dilation aimed at relieving pupillary capture in an undetected reverse position of the sulcus IOL. The pupillary block was successfully treated with 2% pilocarpine and laser peripheral iridotomywas performed to prevent recurrence. The patient experienced recurrent pupillary capture with decreased vision in the affected eye. IOL exchange was the definite treatment resulting in improved vision and proper positioning of the IOL.

**Conclusions and importance:**

When the reverse position of sulcus IOL is detected postoperatively, prophylactic laser peripheral iridotomy should be considered to prevent pupillary block particularly when pupillary capture is present. Pharmacologic pupillary dilation should be performed cautiously. Recurrent pupillary capture is possible and IOL repositioning should be considered to prevent further complications.

## 1. Introduction

In general, posterior chamber intraocular lens (PCIOL) implantation is uncommonly related to pupillary block and pupillary capture especially after introducing the posterior angulated haptic IOL and capsulorhexis technique.^[1]^ Sulcus IOL implantation is a common procedure performed in complicated cataract surgery. Evidence has suggested the association between sulcus IOL position and the risk of pupillary block and pupillary capture especially when non-angulated haptics IOL was used.^[2,3]^ Incorrect insertion of IOL in the sulcus has been reported to occur with a frequency of 1.3% even among experienced surgeons.^[4]^ Theoretically, putting reverse IOL in the sulcus is probably problematic. It not only causes a visual problem but also leads to uncommon complications. We present a case of recurrent pupillary capture and pseudophakic pupillary block related to reverse sulcus IOL implantation.

## 2. Case report

**Figure 1:**
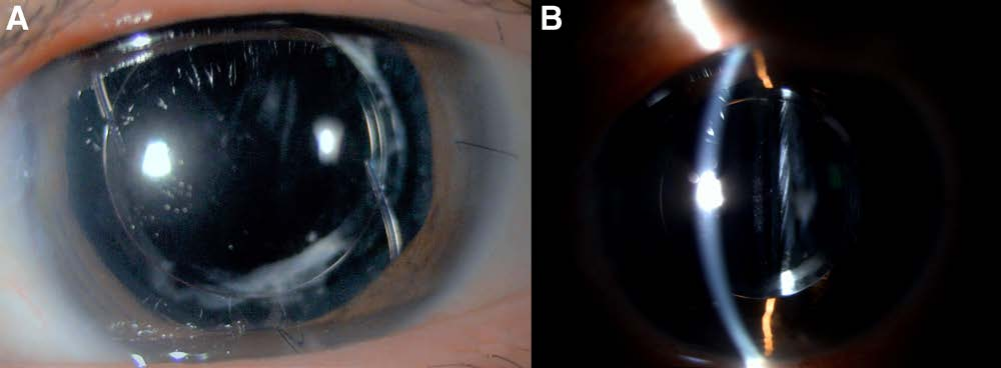
. A) After pupillary dilation, pupillary capture was resolved, and the reverse position of the sulcus intraocular lens was detected. B) The border of the anterior vaulted optic was on the same level as the pupillary plane.

**Figure 2:**
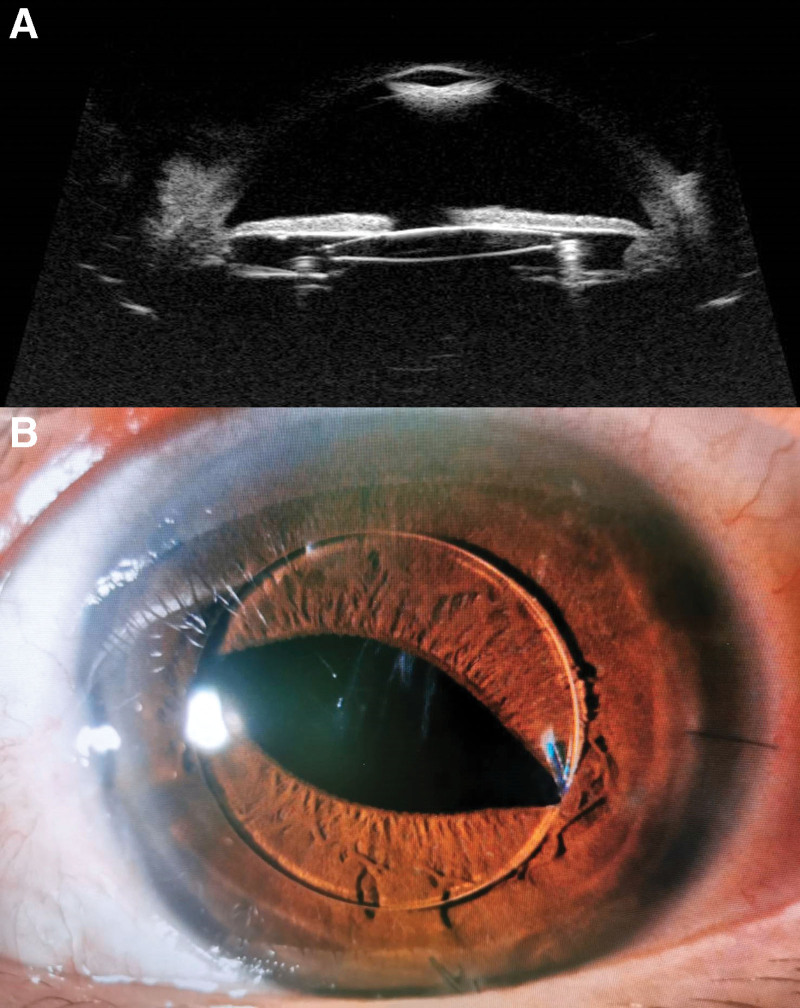
. A) After laser peripheral iridotomy, ultrasound biomicroscopy showed misalignment of the intraocular lens in the sulcus; the optic was anteriorly vaulted and appositioned to the posterior surface of the iris. Deep anterior chamber and widely open-angle were also noted. B) Circumferential pupillary capture was noted 2 weeks later. No evidence of pupillary block was found due to patent iridotomy.

A 44-year-old man attended a follow-up visit after cataract surgery of the left eye. His surgery had been complicated by a posterior capsular tear requiring anterior vitrectomy and implantation of +19.5 diopter Sensar AR40e lens in the sulcus (3-piece, 6 mm biconvex optic, 13 mm length, 5° posteriorly angulated). Ocular examination and visual acuity (VA) were unremarkable earlier. Four weeks postoperatively, the patient reported blurry vision in the left eye for several days prior to the visit. VA was 20/125 improved to 20/32 using a pinhole. Inferior half pupillary capture was found on the slit-lamp examination. Pharmacologic pupil dilation was performed aimed at relieving the pupillary capture. After pupil dilation, slit-lamp examination revealed a reversed position of sulcus IOL and pupillary capture was relieved (Fig. 1). The patient was asked to stay in a supine position and 2% pilocarpine was prescribed to induce miosis which was not successful until office hour was over. The patient was sent home and asked to come back the next day. He was advised to stay in a supine position as much as possible. Six hours later, he came to the emergency room because of sudden pain, blurry vision, and redness in the affected eye. VA was hand motion and intraocular pressure (IOP) was 48 mm Hg. Ciliary injection and cloudy cornea were noted. The pupil was 6 mm in size which circumferentially attached to the IOL optic border resulting in iris bombe and shallow peripheral anterior chamber. He was diagnosed with acute angle-closure from a pupillary block and was treated using 2% pilocarpine. He stayed in a supine position all night then IOP returned to normal, no pupillary capture, and all symptoms were disappeared. Laser peripheral iridotomy (LPI) was scheduled the next morning aiming to prevent recurrence. After laser treatment, an open-angle on gonioscopy was noted. The mesopic pupil was 5.9 mm in the left eye. Two weeks later, routine examination showed 360 degrees pupillary capture without IOP elevation (Fig. 2). The patient also complained of blurry vision in the left eye. VA was 20/160 and open-angle on gonioscopy was noted. The refraction was −2.00 × 1.25 × 30. The patient has undergone IOL exchange with implantation of +19.0 diopter Aurolens SC6530 lens in the sulcus (single-piece, 6.5 mm optic, 13 mm length, 10° posteriorly angulated). Postoperatively, VA was 20/40, quiet anterior chamber, and IOP within the normal range were noted throughout the follow-up period without any medication. The patient reported better vision without fluctuation. The postoperative refraction was −1.00 × 1.25 × 180.

## 3. Discussion

Pupillary capture and pseudophakic pupillary block are uncommon after PCIOL implantation. Reported causative factors of pupillary capture included a shallow anterior chamber, wound leakage, sulcus fixation, non-angulated IOLs, capsular contraction, extracapsular cataract extraction (ECCE) procedure.^[1–3,5]^ Pupillary capture is normally asymptomatic and rarely causes a pupillary block. However, putting PCIOL in a reverse position may lead to different outcomes. Several studies reported pupillary capture and subsequent pupillary block related to reversed IOL in both sulcus and capsular bag.^[6,7]^ Even in uncomplicated PCIOL in the bag, these complications occurred.^[8]^ The authors hypothesized that too large anterior capsulorhexis, postoperative capsular contraction, and IOL model might play a role in these complications.

Dilated fundus examination has been reported to induce pupillary capture and subsequent pupillary block in a patient who had a reversed MA60AC IOL in the capsular bag.^[7]^ IOL was pushed back behind the iris under a slit-lamp using a nasolacrimal canula. No recurrent complications were noted throughout 7 months of follow-up. Our patient presented partial pupillary capture without pupillary block, and the reverse position of IOL was unnoticeable. Therefore, pupillary dilation was performed aiming to reposition the IOL. Pupillary capture was resolved but while the pupil returned to its normal position, it apposed to the reversed optic border leading to the pupillary block. We suggested that pupil dilation is highly cautious in eyes with reverse IOL position. However, this condition was successfully treated using LPI.

Reversed sulcus IOL implantation can lead to chronic angle-closure glaucoma as a result of an alteration of the anterior chamber angle.^[6]^ Their patient presented chronic angle-closure glaucoma and acute pupillary block many years after cataract surgery. Extensive peripheral anterior synechiae was found and combined surgery was needed to control IOP. Although our patient had a deep anterior chamber and widely open-angle even having reversed IOL, the anterior chamber was deeper after IOL exchange (3.46 and 4.18 mm, respectively). We anticipate that this difference may have a significant effect on susceptible subjects.

Currently, most IOLs are manufactured in a biconvex design and the refractive shift owing to a reversed optic alone is nil. The AR40e model has an anterior biconvex optic which has been reported to have no significant effect on refraction when reversed.^[9]^ However, the forward displacement of the optic induced by angulated haptics can induce a myopic refractive shift. One study focused on trigonometric calculations assessing the extent of forwarding movement of the reversed optics and the refractive power of the eyes. Various degrees of a myopic shift were found related to IOL length and haptic angle.^[10]^ This information was confirmed by improved vision and refractive error after the surgery in our patient.

Because of a large pupil size in dim light reported on mesopic pupil measurement, we chose a new IOL with a larger optic diameter and high angulated haptics to prevent pupillary capture. Evidence showed the association between sulcus IOL and uveitis glaucoma hyphema syndrome (UGH) in the presence of reverse pupillary block.^[11]^ The authors found a resolution of UGH syndrome after LPI in all eyes. We postulate that this serious complication could also occur in our patient. Misalignment of anterior vaulted IOL and apposition between IOL and posterior surface of the iris shown in ultrasound biomicroscopy increased the risk of UGH syndrome.

## 4. Conclusions

To prevent pupillary capture and pseudophakic pupillary block, immediate IOL repositioning is recommended when a reverse position of sulcus IOL is detected intraoperatively. When the condition is detected postoperatively, prophylactic LPI should be considered to prevent pupillary block particularly when pupillary capture is present. Pharmacologic pupillary dilation should be performed cautiously. IOL repositioning should be considered to prevent UGH syndrome and progressive angle closure in susceptible eyes. Improved vision is beneficial in some cases.

## 5. Patient consent

The patient already discussed information of the study with the principal author and provided his written informed consent to publication. This study was approved by the Institutional Review Board (IRB), Royal Thai Army Medical Department. IRB approval number was 1240/2564.

## Author contributions

**Conceptualization:** Panrapee Funarunart, Isaraporn Treesit.

**Data curation:** Panrapee Funarunart.

**Funding acquisition:** Panrapee Funarunart.

**Investigation:** Panrapee Funarunart.

**Methodology:** Panrapee Funarunart.

**Project administration:** Isaraporn Treesit.

**Resources:** Isaraporn Treesit.

**Software:** Panrapee Funarunart.

**Supervision:** Isaraporn Treesit.

**Validation:** Panrapee Funarunart.

**Visualization:** Panrapee Funarunart, Isaraporn Treesit.

**Writing – original draft:** Panrapee Funarunart.

**Writing – review & editing:** Isaraporn Treesit.
